# Orthodontic Extrusion in Daily Clinical Practice: Management of Fractured or Damaged Anterior Teeth

**DOI:** 10.3390/jpm15090408

**Published:** 2025-09-01

**Authors:** Giuseppina Malcangi, Grazia Marinelli, Maral Di Giulio Cesare, Sharon Di Serio, Marialuisa Longo, Andrea Carbonara, Francesco Inchingolo, Alessio Danilo Inchingolo, Ioana Roxana Bordea, Andrea Palermo, Angelo Michele Inchingolo, Gianna Dipalma

**Affiliations:** 1 Interdisciplinary Department of Medicine, University of Bari “Aldo Moro”, 70124 Bari, Italy; giuseppinamalcangi@libero.it (G.M.); graziamarinelli@live.it (G.M.); maral.digiuliocesare@uniba.it (M.D.G.C.); sharon.diserio@uniba.it (S.D.S.); marialuisa.longo@uniba.it (M.L.); andrea.carbonara@libero.it (A.C.); francesco.inchingolo@uniba.it (F.I.); angelomichele.inchingolo@uniba.it (A.M.I.); gianna.dipalma@uniba.it (G.D.); 2 Department of Oral Rehabilitation, Faculty of Dentistry, University of Medicine and Pharmacy Iuliu Hatieganu, 400012 Cluj-Napoca, Romania; 3 Department of Experimental Medicine, University of Salento, 73100 Lecce, Italy; andreapalermo@unisalento.it

**Keywords:** extrusion, orthodontics, damaged teeth, fractured teeth

## Abstract

**Background**. Orthodontic extrusion (OE), or forced eruption, is a conservative technique used to recover teeth affected by coronal fractures, traumatic intrusions, or severe caries. It involves applying light, continuous forces to induce vertical tooth movement, promoting tissue remodeling through periodontal ligament stimulation. **Materials and Methods**. This narrative review included studies investigating OE as a therapeutic approach for the management of deep or subgingival carious lesions, traumatic dental injuries (such as intrusion or fracture), or for alveolar ridge augmentation in implant site development. OE is typically performed using fixed appliances such as the straight-wire system or, in selected cases, clear aligners. Forces between 30 and 100 g per tooth are applied, depending on the clinical situation. In some protocols, OE is combined with fiberotomy to minimize gingival and bone migration. **Results**. Studies show that OE leads to significant vertical movement and increases in buccal bone height and interproximal septa. It enhances bone volume in targeted sites, making it valuable in implant site development. Compared to surgical crown lengthening, OE better preserves periodontal tissues and improves esthetics. **Conclusions**. In this narrative review is analized how OE is effective for managing traumatic intrusions and compromised periodontal sites, particularly when paired with early endodontic treatment. It reduces the risks of ankylosis and root resorption while avoiding invasive procedures like grafting. Although clear aligners may limit axial tooth movement, OE remains a minimally invasive, cost-effective alternative in both restorative and implant dentistry.

## 1. Introduction

Preserving compromised teeth, particularly in the anterior region, is one of the most complex and important challenges in modern restorative and multidisciplinary dentistry. In the era of “minimally invasive and personalized dentistry”, clinical decisions must be guided by conservative principles, aiming to maintain the natural dentition whenever possible.

This reflects the principles of personalized medicine, where treatment plans are tailored to the individual biological and clinical characteristics of the patient to optimize outcomes. In this clinical context, orthodontic extrusion, also known as “forced orthodontic eruption”, is configured as a predictable and effective technique for the recovery of severely compromised teeth, allowing the restoration of a sufficient quantity of coronal and periodontal tissue useful for functional and esthetic rehabilitation.

Orthodontic extrusion consists of the application of light and continuous forces aimed at causing the coronal migration of the tooth inside the alveolus, through a tissue remodeling process that involves both the periodontal ligament and the alveolar bone. Controlled extrusion, originally developed for orthodontic management of dental inclusions and impactions, has become a key technique in endodontics and prosthetics, offering a viable alternative to periodontal surgery or tooth extraction.

Anterior teeth, due to their esthetic and functional importance, are often the subject of particular attention in conservative treatment strategies. However, dental trauma, extensive subgingival caries, coronal root fractures, or iatrogenic complications can seriously compromise the residual tooth structure, making it difficult to obtain a correct marginal seal and a sufficient prosthetic ferrule. In the absence of an adequate residual coronal height, the creation of indirect restorations such as full crowns is clinically questionable, both for biomechanical reasons and for the risk of carious recurrences or prosthetic fractures.

In these cases, orthodontic extrusion is proposed as an auxiliary technique that allows bringing to the surface a portion of the healthy and intact dental root, which would otherwise remain inaccessible for the preparation of the prosthetic margin [1,2,3,4]. This technique respects the biological concept of “biologic width” or “biological space”, avoiding tissue violations that could lead to chronic inflammation, gingival retraction, and loss of periodontal attachment, thereby aligning with personalized biological considerations for each patient [5,6,7,8,9,10,11,12,13,14]. At the same time, extrusion stimulates the formation of new bone and connective tissue, contributing to the regeneration of supporting tissues and to the improvement of the peri-prosthetic tissue profile [15,16,17,18,19]. This highlights a regenerative strategy based on the patient’s intrinsic biological capacity, illustrating principles of personalized regenerative medicine.

From a biomechanical point of view, orthodontic extrusion must be performed with extreme attention to the control of the applied forces, which must be slow and constant, generally between 15 and 60 g, in order to avoid root resorption or compromise of the support apparatus [20,21,22,23]. Moreover, in certain cases, adjunctive fibrotomy procedures or supplementary gingival and osseous surgeries are required to inhibit coronal migration of soft and hard tissues, thereby preserving the vertical height gained [24,25,26,27]. Orthodontic devices that can be used for extrusion include:

- Traditional brackets with light alloy arches and vertical levers.
- Segmented arches with NiTi springs or TMA wires.
- Auxiliary anchors such as mini-orthodontic screws (TADs), which allow precise control and reduce dependence on adjacent anchorage elements.
- Transparent aligners, which, with appropriate “attachments” and digital planning, can generate programmed extrusive movements.
- Mixed systems, with prosthetic aids (e.g., glued caps or buttons) to facilitate direct traction of the element to be extruded [28,29,30,31].

The choice of technique is guided by a personalized assessment of the clinical situation, patient compliance, and overall treatment goals, embodying the tailored treatment concept of personalized medicine [32,33,34,35]. Regardless of the technique used, the subsequent “stabilization” or containment phase is crucial for maintaining the results obtained and requires a period of immobilization of the extruded element that can vary from 4 to 8 weeks [36,37,38,39,40,41].

Orthodontic extrusion finds its main indication in the following clinical conditions:

- Subgingival coronal fractures, especially in the anterior sectors, in which it is desired to preserve the tooth rather than resort to implants [42,43,44,45].
- Destructive caries below the gingival level, which prevents the creation of a correct coronal restoration [46,47,48].
- Endodontic lesions with coronal resorption of the root but preservation of the root apex.
- Orthodontic preparation for deferred extraction, in which extrusion allows regeneration of bone tissue and facilitates subsequent implant positioning in more favorable conditions [49,50,51].

In the anterior region, esthetics play a crucial role. Preserving the interdental papilla, maintaining a harmonious gingival profile, and seamlessly blending the final restoration with the adjacent teeth are essential factors for therapeutic success [52,53,54,55,56,57,58,59,60,61,62]. Orthodontic extrusion, which helps avoid extensive surgical flaps and preserves the integrity of periodontal structures, is among the most commonly used orthodontic movements and often represents the most conservative and esthetically predictable approach [63,64,65,66,67,68,69,70]. This conservative biological approach aligns with translational medicine by applying fundamental biological principles to clinical practice. Additionally, extrusion enables natural regeneration of coronal alveolar bone without the need for guided regeneration or grafting techniques [71,72,73,74,75,76,77]. In fact, during the extrusive orthodontic movement, the applied traction stimulates osteoblastic activity in the coronal direction, with progressive apposition of bone along the periodontal ligament [78,79,80,81,82,83,84]. This well-documented phenomenon is especially beneficial in young patients, where tissue quality and regenerative capacity are optimal. However, the successful application of the technique demands close interdisciplinary collaboration among orthodontists, prosthodontists, endodontists, and occasionally periodontists to ensure precise planning and seamless execution [85,86,87,88]. This interdisciplinary strategy exemplifies the translational medicine model by integrating various specialties to tailor patient care. Clear communication with the patient regarding the longer treatment duration compared to extraction and implant placement is crucial, as the extrusion approach often yields superior long-term clinical outcomes. Nonetheless, orthodontic extrusion presents certain limitations and contraindications [89,90,91,92,93,94,95]. These include: the inability to achieve stable anchorage in patients with a reduced dental arch, the presence of very short or vertically fractured roots, poor patient compliance, and the presence of systemic factors that limit bone healing or orthodontic response [96,97,98,99,100,101,102]. The frequent use of orthodontic extrusion in daily dental practice underscores the need for a narrative review. The goal of this narrative review is to summarize the existing literature on OE and provide a clinical synthesis of its therapeutic applications. This technique enables highly personalized treatment planning, which relies on comprehensive assessments—including medical history, clinical examination, and 3D imaging (CBCT)—to evaluate case suitability and predict outcomes, with particular emphasis on residual bone thickness and root morphology, in alignment with personalized medicine principles [103,104,105,106,107,108,109].

## 2. Materials and Methods

### 2.1. Search Processing

In order to explore the clinical relevance and therapeutic applications of orthodontic extrusion, we conducted a broad literature search across major scientific databases.

Review Registry procedures (full ID: 1059711).

Our approach favored a narrative synthesis, aiming to provide a comprehensive overview rather than a quantitative analysis.

The search included publications from the last 15 years (January 2010 to February 2025), focusing on peer-reviewed articles in English. The databases consulted were PubMed, Web of Science, and Scopus, selected for their wide coverage of biomedical literature. 

The following Boolean keywords were applied: “A”: extrusion”, “B”: orthodont”.

Article-screening strategy

KEYWORDS: “A”: extrusion”, “B”: orthodont”

Boolean Indicators: “A” AND “B”

Timespan: 1 January 2010 to 1 February 2025

Electronic databases: PubMed; Scopus; Web of Science.

The search terms were designed to capture studies discussing orthodontic extrusion in clinical contexts, especially where it was used for the management of deep or subgingival dental lesions.

### 2.2. Inclusion and Exclusion Criteria

For the purposes of this narrative review, studies were included based on their relevance to clinical practice and their contribution to the understanding of orthodontic extrusion as a therapeutic strategy.

Particularly, we considered studies where orthodontic extrusion was used to manage conditions such as subgingival fractures, deep carious lesions, traumatic intrusion, or to aid in implant site development. The review focused on articles that reported clinical outcomes, especially those supported by radiographic or CBCT evaluations.

Rather than strictly applying inclusion and exclusion filters, the selection was guided by clinical relevance and the potential of each article to contribute to a nuanced understanding of the topic.

Although not structured through a formal PICO model, the review was shaped by a central clinical question: how effective is orthodontic extrusion, compared to other approaches, in preserving hard and soft tissues and facilitating long-term rehabilitation in compromised dental situations?

### 2.3. Data Processing

The selected literature was analyzed narratively, focusing on key clinical insights, reported outcomes, and recurring themes across different therapeutic approaches.

Rather than using formal quality assessment tools, the studies were selected and discussed among the authors based on thematic relevance and clinical applicability. Key findings were grouped and interpreted to highlight trends, clinical challenges, and areas in need of further exploration.

## 3. Results

A total of 2337 papers were obtained from the databases Web of Science (554), PubMed (277), and Scopus (1506). This resulted in 1777 articles after eliminating duplicates (560). 120 articles have been eliminated as a systematic review, 30 as in vitro studies and 1616 because off topic or not strictly relevant. This study presents the qualitative analysis of 11 selected articles. “In accordance with PRISMA guidelines and the systematic review standards, the included studies are described in terms of their design, objectives, materials and methods, and principal findings. This structured approach ensures methodological transparency and supports the interpretation of the heterogeneity across study designs.”

### Methodological Considerations and Study Quality

While this review does not adopt a formal risk of bias assessment, we considered the methodological soundness of each study by examining aspects such as study design, sample size, clarity of outcome measures, and presence or absence of control groups.

Randomized trials were generally more robust in methodology, especially when randomization and blinding were clearly described. Observational studies, while offering valuable clinical insight, often showed limitations related to sample size or reporting transparency.

Notably, certain studies—such as those by G. Rossini et al. (2021) [110,111], I. Arsić et al. (2022) [112], and J. Zhang et al. (2025) [113]—exhibited strong methodological design with consistent and well-reported outcomes. Conversely, some case series, like those by Bruhnke et al. and Shalish et al., presented concerns mainly related to attrition and performance bias, which may affect data interpretation.

These observations underscore the importance of interpreting results in light of methodological limitations. While most included studies contribute meaningfully to the understanding of orthodontic extrusion, issues such as incomplete data, limited participant numbers, and the absence of control groups remain common barriers to evidence strength.

Overall, these findings suggest that while the majority of the studies are methodologically sound, certain limitations, such as small sample sizes, incomplete data handling, and absence of control groups, should be carefully considered when interpreting the conclusions regarding the effectiveness and safety of orthodontic extrusion techniques.

Orthodontic extrusion, also referred to as forced eruption, has increasingly gained recognition as a conservative, biologically driven, and interdisciplinary tool in modern dental practice [114]. It is indicated in a wide range of clinical scenarios, including the treatment of traumatic dental intrusion, the management of subgingival and subcrestal fractures, the rehabilitation of severely decayed teeth, and even the preparation of future implant sites [115,116,117,118,119]. Its importance is especially evident in the anterior region, where both esthetic and functional requirements are paramount, and where traditional surgical crown lengthening often proves inadequate or overly invasive [120]. By contrast, orthodontic extrusion provides a minimally invasive alternative that leverages the body’s natural biological response to controlled mechanical forces [113]. Through activation of the periodontal ligament and alveolar bone, it promotes coronal migration of both hard and soft tissues, allowing for the re-establishment of the biologic width and sufficient tooth structure for prosthetic rehabilitation while preserving gingival architecture [121,122,123,124,125,126]. This principle of tissue engineering through orthodontic movement exemplifies translational medicine, where fundamental biological mechanisms are applied in clinical settings to improve outcomes [127,128,129,130,131,132].

Numerous studies have investigated the clinical and biological effects of orthodontic extrusion [133]. Arsić et al. conducted a prospective clinical study using CBCT to evaluate changes in the alveolar bone following orthodontic extrusion [134,135,136,137,138]. Their findings showed a mean vertical tooth movement of 1.52 mm, accompanied by significant increases in buccal bone plate and mesial interproximal septum height, with minimal palatal bone change [139]. Importantly, although some degree of apical root resorption was observed, it was considered clinically insignificant [140,141,142,143,144]. These results support the hypothesis that orthodontic extrusion can stimulate localized bone apposition in the direction of tooth movement, particularly when the periodontal ligament remains intact and functional [111,145,146,147]. Furthermore, the pattern and magnitude of tissue remodeling appear closely correlated with the direction and extent of extrusion, reinforcing the importance of personalized treatment protocols tailored to individual anatomical responses [148]. Bruhnke and colleagues emphasized the relevance of orthodontic extrusion in restoring severely compromised teeth, particularly those with subgingival lesions, noting that the presence of sufficient residual tooth structure and a proper ferrule are critical for long-term success [110]. While surgical crown lengthening may still be a viable option in posterior teeth, in the anterior esthetic zone, orthodontic extrusion is often the more favorable approach due to its tissue-preserving nature and more predictable esthetic results [149].

Economically, the technique also shows promise. A health economic evaluation conducted at Charité–Universitätsmedizin Berlin analyzed the cost-effectiveness of orthodontic extrusion followed by prosthetic rehabilitation in cases involving deep subgingival defects [150]. The study, which included 35 teeth, reported a 91% survival rate and an 83% clinical success rate over an average follow-up of 49 .months. While the median cost per case was approximately €2284, complications such as tooth loss significantly increased the total expenditure [151,152,153,154,155]. These findings suggest that orthodontic extrusion, when properly indicated and executed, may offer not only clinical reliability but also long-term economic sustainability, particularly as a tooth-preserving alternative to extraction and implant placement [156,157,158,159,160].

Clinical protocols vary depending on the intended outcome and the tissue response. Uravić Crljenica et al. proposed a rapid forced orthodontic extrusion (FOE) technique combined with regular fiberotomy to prevent undesirable migration of gingival and osseous tissues [161,162,163,164,165]. In their study involving 10 premolars with subgingival lesions, extrusion averaged 2 mm, with about 49% of the movement reflected in soft tissue migration and 18% in bone displacement [166,167,168,169,170]. While effective, this approach also highlighted the partial and somewhat unpredictable behavior of surrounding tissues during rapid extrusion, further supporting the value of customizing extrusion speed and timing. Similarly, Ao Sun et al. presented a digital extrusion protocol using CAD-CAM fabricated pontics and elastic traction, which enabled precise planning and improved esthetics during treatment [171,172,173,174,175]. This method proved successful in managing single-tooth fractures with minimal impact on adjacent structures [145,176,177,178,179,180]. Another case series by Bosly showcased the use of customized appliances and extrusion protocols in young female patients with severely damaged anterior teeth, demonstrating favorable radiographic bone preservation and high patient satisfaction, all while avoiding invasive surgery [181,182,183,184,185,186].

In the context of dental trauma, especially traumatic intrusion, orthodontic extrusion has proven particularly valuable. Shalish et al. compared traumatized intruded teeth with non-traumatized controls and found that all intruded teeth lost vitality, and 90% developed pulp necrosis, frequently accompanied by complications such as root resorption and canal obliteration. When spontaneous re-eruption does not occur, orthodontic extrusion becomes the treatment of choice, albeit with careful planning to reduce iatrogenic damage, particularly in teeth with mature apices. Early endodontic treatment is often necessary [187,188,189,190,191,192,193]. A case by Hayriye Sönmez et al. reported on the successful orthodontic extrusion of an intruded central incisor in an 8-year-old girl following failed spontaneous eruption, with pulp canal obliteration observed after five years but continued functionality. Zhang et al., in a prospective clinical study, evaluated 28 intruded teeth treated with extrusion and 29 controls managed with spontaneous re-eruption. The success rate for extrusion reached 96.4%, slightly higher than the 90% observed in the spontaneous eruption group [194,195,196,197]. Despite a high incidence of pulp necrosis, especially in mature teeth, root growth continued in immature teeth. The risk of replacement resorption (ankylosis) was low and may even be reduced by early application of orthodontic force. These results confirm that controlled extrusion, when carefully timed and biologically guided, offers a safe and effective alternative to passive observation or surgical repositioning in cases of dental intrusion.

From a biomechanical perspective, extrusion using clear aligners has been the focus of several investigations, particularly for open bite correction. Rossini and Modica performed finite element analyses (FEA) to assess the behavior of aligners during planned upper incisor extrusion. Their findings showed that bodily incisor extrusion is difficult to achieve with aligners alone, due to associated retroclination, lateral flaring, and tipping of adjacent teeth [198,199,200,201,202]. Attachments on both anterior and posterior teeth improved force control and anchorage, enhancing predictability. Groody et al. [120] corroborated these results, noting that most vertical correction with aligners occurs through mandibular counterclockwise rotation and molar intrusion rather than true incisor extrusion. These biomechanical insights highlight the need for improved treatment planning and appliance design when vertical movement is required, particularly in aligner-based therapy.

**Figure 1:**
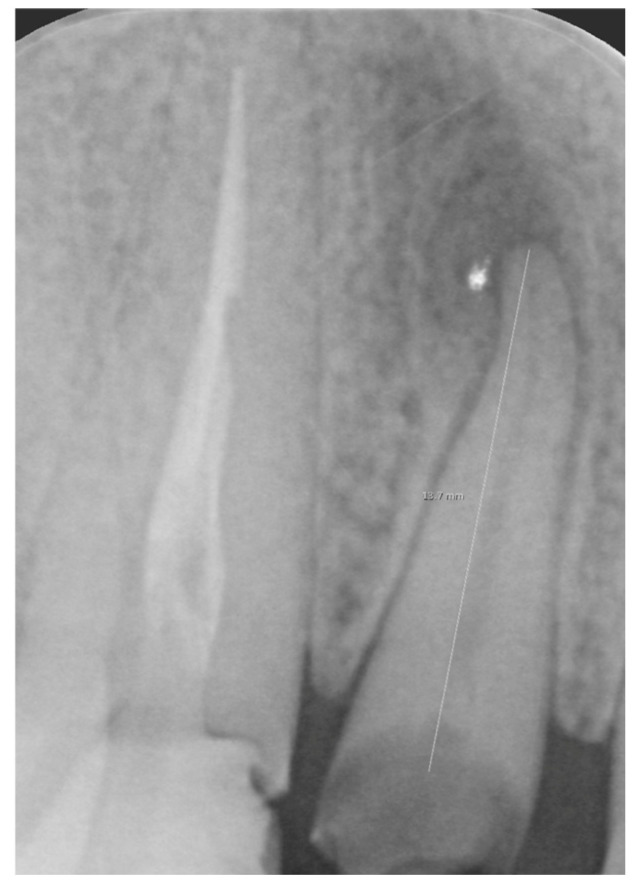
Intraoral x-ray of a tooth with a destructive carious lesion.

Lastly, Borrelli De Barros presented a compelling clinical case of a 56-year-old woman with severe periodontal bone loss in the anterior maxilla. Following comprehensive periodontal therapy, slow orthodontic extrusion was performed over 14 months to increase vertical bone height [203,204,205,206,207]. This biologically driven extrusion allowed implant placement without grafting, followed by immediate provisional crowns for soft tissue contouring. At six-year follow-up, the patient exhibited stable function and excellent esthetics, demonstrating how forced eruption can be successfully integrated into a regenerative, multidisciplinary treatment pathway [112]. Overall, orthodontic extrusion offers a biologically sound, minimally invasive, and cost-effective treatment option across a range of clinical indications. Its success relies heavily on personalized protocols, interdisciplinary collaboration, and an in-depth understanding of the biological principles that govern tissue remodeling, positioning it as a key tool in the modern, patient-centered dental approach (Figure 1).

## 4. Conclusions

In conclusion, orthodontic extrusion (OE), as reviewed in the current literature, stands as a conservative and clinically effective tool for managing compromised anterior teeth and constitutes a versatile and minimally invasive technique with profound clinical significance. Whether applied for the rehabilitation of traumatically intruded teeth, the restoration of teeth exhibiting subgingival defects or fractures, or for the preparation of implant sites, OE confers numerous advantages. Its ability to enhance bone volume, especially in the buccal and mesial regions, makes it an indispensable tool in both restorative dentistry and periodontics. Furthermore, OE maintains periodontal health, preserves natural tooth structure, and avoids the complications often associated with more invasive surgical procedures. While the technique shows promising long-term clinical outcomes, it requires careful planning, proper execution, and collaboration between orthodontists, periodontists, and prosthodontists. Cost-effectiveness studies and biomechanical analyses indicate that, when applied appropriately, orthodontic extrusion (OE) represents both an efficient and economical treatment modality, rendering it a compelling alternative to implant therapy in selected cases. Nonetheless, it remains imperative to further advance our understanding of OE’s effects on root stability, soft tissue management, and bone remodeling to ensure its optimal clinical application. Consequently, orthodontic extrusion constitutes a vital instrument in contemporary dental practice, facilitating predictable, functional, and esthetically favorable outcomes. This review consolidates findings from existing clinical studies to guide clinicians in adopting OE where appropriate, ensuring personalized, evidence-based care.

## Data Availability

Not applicable.

## Abbreviations

**Abbreviations**	**Definition**
OE	Orthodontic Extrusion
FOE	Forced Orthodontic Extrusion
CBCT	Cone Beam Computed Tomography
PRISMA	Preferred Reporting Items for Systematic Reviews and Meta-Analyses
PDL	Periodontal Ligament
TADs	Temporary Anchorage Devices
CAD-CAM	Computer-Aided Design/Computer-Aided Manufacturing
